# Supplementary material for: A Critical Review of Methods for the Quantification of Phenotypic Variation, with Examples from Canis and Sigmodontine Rodents
Source: Integr Org Biol. 2026 Oct 1;8(1):obag059. doi: 10.1093/iob/obag059 (PMC13714550; doi:10.1093/iob/obag059)
Supplement: Supplementary file 1 [file obag059_supplemental_files.zip › IOB_2026_064_Code1.docx]

PermTestBoot.R

okeefef

2026-02-02

```
#R Code for Phenotypic Hyperellipse Geometry, 2.
#Code for bootstrapping Effective Rank, Decay Slope,
#Rank Compression Ratio, and Regression Slope Ratio
# F. Robin O'Keefe 2026

#attach necessary libraries

library(dplyr)
##
## Attaching package: 'dplyr'
## The following objects are masked from 'package:stats':
##
##     filter, lag
## The following objects are masked from 'package:base':
##
##     intersect, setdiff, setequal, union
library(broom)
library(readxl)

#Define a variable containing the GPA X,Y landmark data. The data is read in as a data frame from an Excel file.

GPALMData <- read_excel("~/Desktop/DimensionlessStuff/OryAllAllom.xlsx")
#GPALMData <- read_excel("~/Desktop/DimensionlessStuff/Nectomys69.xlsx")
#GPALMData <- read_excel("~/Desktop/DimensionlessStuff/Oryzomys_p69.xlsx")
#View(GPALMData)

SampleN<-nrow(GPALMData)

#initialize variables

DdBoot=matrix(nrow=1,ncol=0)
PermRankBoot=matrix(nrow=1,ncol=0)
CompressionBoot=matrix(nrow=1,ncol=0)
SlopeBoot=matrix(nrow=1,ncol=0)
EffRankBoot=matrix(nrow=1,ncol=0)
SumVar=matrix(nrow=1,ncol=0)
Vrel26=matrix(nrow=1,ncol=0)

#Start bootstrap loop
for (j in 1:1000){
  bootsample=sample_n(GPALMData, SampleN, replace = TRUE)
  PermSlope=matrix(nrow=1,ncol=0)
#calculate initial parameters
iCVLM= cov(bootsample)

iCVLMeigen=eigen(iCVLM)

iEigenVal<-iCVLMeigen$value

iEigenSum=sum(iEigenVal)

iPofK<-iEigenVal/iEigenSum

ilnPofK<-log(iPofK)

iProduct<-ilnPofK*iPofK

iSumProduct<-sum(iProduct, na.rm=TRUE)

iShannonEntropy <- -1*iSumProduct

iEffectiveRank<-exp(iShannonEntropy)

EffRank=matrix(nrow=1,ncol=0)

ERInteger<-as.integer(iEffectiveRank)
ERRoundUp= ERInteger+1
EigenTrim<-iEigenVal[1:ERRoundUp]


#Start permutation loop
for (i in 1:500){

  #Permute rows in each variable column vector independently for permutation test.

  PermLM = apply(bootsample, 2, sample, SampleN, replace=FALSE)
                                  #sample size

  #Define variable that is the covariance matrix of the above. Be sure to specify only the landmark columns in the data frame.
  CVLM= cov(PermLM)
  #toggle for cov/cor

  #Define object that is the eigenanalysis of the covariance matrix.
  CVLMeigen=eigen(CVLM)

  EigenVal<-CVLMeigen$values

  #Define the summation of eigenvalues from the eigenanalysis.
  EigenSum=sum(EigenVal)

  #Create vector of scaled eigenvalues
  PofK<-EigenVal/EigenSum

  #Creat vector of lnPofK
  lnPofK<-log(PofK)

  Product<-lnPofK*PofK

  SumProduct<-sum(Product, na.rm=TRUE)

  ShannonEntropy <- -1*SumProduct

  EffectiveRank<-exp(ShannonEntropy)

  EffRank <-cbind(EffRank, EffectiveRank)

  lnEigenVal<-log(EigenVal)

  RankE<-seq(from=1,to=50,by=1)

  Line<-lm(lnEigenVal[1:50] ~ RankE)

  Slope=coef(Line)[2]
  PermSlope<-cbind(PermSlope, Slope)

}

SumVar<-cbind(SumVar,iEigenSum)
PermRankBoot<-cbind(PermRankBoot, mean(EffRank))

#calculate and print st deviation and mean for each parameter
RankCompression<-(mean(EffRank)/iEffectiveRank)

EffRankBoot<-cbind(EffRankBoot,iEffectiveRank)
CompressionBoot<-cbind(CompressionBoot,RankCompression)

#Begin Vrel calculation
EigenTrim<-iEigenVal[1:26]
meanEigen<-(sum(EigenTrim))/26
diffEigen<-(EigenTrim)-meanEigen
sqdiffEigen<-(diffEigen)^2
SumSqDifEig<-sum(sqdiffEigen)
VaRel<-(SumSqDifEig)/(((meanEigen)^2)*650)
Vrel26<-cbind(Vrel26,VaRel)

#Begin eigenvalue regression
#create ordinal rank vector of length EigenTrim
RankE<-seq(from=1, to=length(EigenTrim), by=1)

# ln lambda
lnEigenTrim<-log(EigenTrim)

# regression
LineFit<-lm(lnEigenTrim ~ RankE)

NormSlope<- coef(LineFit)[2]/mean(PermSlope)

SlopeBoot<-cbind(SlopeBoot,NormSlope)
cat("\014")
print (j)
}
## [1] 1…
## [1] 1000
cat("Bootstrapped Sum of Variance: ", mean(SumVar))
## Bootstrapped Sum of Variance:  0.001246203
cat("   SD: ", sd(SumVar))
##    SD:  4.626877e-05
cat("Bootstrapped Effective Rank: ", mean(EffRankBoot))
## Bootstrapped Effective Rank:  19.60973
cat("   SD: ", sd(EffRankBoot))
##    SD:  1.015615
cat("Bootstrapped Permuted Effective Rank: ", mean(PermRankBoot))
## Bootstrapped Permuted Effective Rank:  43.01121
cat("   SD: ", sd(PermRankBoot))
##    SD:  0.557858
cat("Bootstrapped Rank Compression Ratio, piRe/Re: ", mean(CompressionBoot))
## Bootstrapped Rank Compression Ratio, piRe/Re:  2.19864
cat("   SD: ", sd(CompressionBoot))
##    SD:  0.117583
cat("Bootstrapped Vrel26 :", mean(Vrel26))
## Bootstrapped Vrel26 : 0.04131769
cat("   SD :", sd(Vrel26))
##    SD : 0.004611842
cat("Bootstrapped Slope Ratio (Slope/Permuted Slope) :", mean(SlopeBoot))
## Bootstrapped Slope Ratio (Slope/Permuted Slope) : 2.495088
cat("   SD :", sd(SlopeBoot))
##    SD : 0.08295543
```
