# Supplementary material for: A Critical Review of Methods for the Quantification of Phenotypic Variation, with Examples from Canis and Sigmodontine Rodents
Source: Integr Org Biol. 2026 Oct 1;8(1):obag059. doi: 10.1093/iob/obag059 (PMC13714550; doi:10.1093/iob/obag059)
Supplement: Supplementary file 1 [file obag059_supplemental_files.zip › IOB_2026_064_Code2.docx]

PermTestPlot.R

okeefef

2026-02-02

```
#R Code for Phenotypic Hyperellipse Geometry, 1.
#Code for calculating and plotting Effective Rank, Decay Slope,
#Rank Compression Ratio, and Regression Slope Ratio
# F. Robin O'Keefe 2026

#attach necessary libraries

library(dplyr)
##
## Attaching package: 'dplyr'
## The following objects are masked from 'package:stats':
##
##     filter, lag
## The following objects are masked from 'package:base':
##
##     intersect, setdiff, setequal, union
library(broom)
library(readxl)
library(geomorph)
## Loading required package: RRPP
## Loading required package: rgl
## Loading required package: Matrix
#Define a variable containing the GPA X,Y landmark data.
#The data is read in as a data frame from an Excel file.

GPALMData <- read_excel("~/Desktop/DimensionlessStuff/NectoAllAllom.xlsx")
#GPALMData <- read_excel("~/Desktop/DimensionlessStuff/MicroALlAllom.xlsx")
#GPALMData <- read_excel("~/Desktop/DimensionlessStuff/Nectomys69.xlsx")
#GPALMData <- read_excel("~/Desktop/DimensionlessStuff/OryAllAllom.xlsx")

#View(GPALMData)

SampleN<-nrow(GPALMData)

EffRank=matrix(nrow=1,ncol=0)
DdBoot=matrix(nrow=1,ncol=0)
PermSlope=matrix(nrow=1,ncol=0)

#calculate initial parameters
iCVLM= cov(GPALMData)

iCVLMeigen=eigen(iCVLM)

iEigenVal<-iCVLMeigen$value

iEigenSum=sum(iEigenVal)

iPofK<-iEigenVal/iEigenSum

ilnPofK<-log(iPofK)
## Warning in log(iPofK): NaNs produced
iProduct<-ilnPofK*iPofK

iSumProduct<-sum(iProduct, na.rm=TRUE)

iShannonEntropy <- -1*iSumProduct

iEffectiveRank<-exp(iShannonEntropy)

cat("Effective Rank: ",iEffectiveRank)
## Effective Rank:  20.09512
ERInteger<-as.integer(iEffectiveRank)
ERRoundUp= ERInteger+1
EigenTrim<-iEigenVal[1:ERRoundUp]

#Plot scree plot

RankE<-seq(from=1,to=50,by=1)

plot (RankE,iEigenVal[1:50], cex = 1.5, pch=16,main="Raw Scree Plot")
abline(v=iEffectiveRank, lty = 2, col="black",lwd=2)
```

plot (RankE,iPofK[1:50], cex = 1.5, pch=16, ylim = c(0,0.30), main="Percent Scree Plot")
abline(v=iEffectiveRank, lty = 2, col="black",lwd=2)

```
par(xaxs = "i", yaxs = "i")
plot (x = 1,
     xlab = "Rank",
     ylab = "ln lambda",
     xlim = c(1, 50),
     ylim = c(-14, -7),
     xaxp = c(1, 50, 49),  cex.axis=1.5,
     main = "Ln Lambda vs. Rank",
     type = "n")
     points (x=RankE, y=log(iEigenVal[1:50]),
     cex=1.5,
     pch=16)
#Start permutation loop
for (i in 1:5000){

  #Permute rows in each variable column vector independently for permutation test.

  PermLM = apply(GPALMData, 2, sample, SampleN, replace=FALSE)
                                    #sample size

  #Define variable that is the covariance matrix of the above. Be sure to specify only the landmark columns in the data frame.
  CVLM= cov(PermLM)
  #toggle for cov/cor

  #Define object that is the eigenanalysis of the covariance matrix.
  CVLMeigen=eigen(CVLM)

  EigenVal<-CVLMeigen$values

  #Define the summation of eigenvalues from the eigenanalysis.
  EigenSum=sum(EigenVal)

  #Create vector of scaled eigenvalues
  PofK<-EigenVal/EigenSum

  #Creat vector of lnPofK
  lnPofK<-log(PofK)

  Product<-lnPofK*PofK

  SumProduct<-sum(Product, na.rm=TRUE)

  ShannonEntropy <- -1*SumProduct

  EffectiveRank<-exp(ShannonEntropy)

  EffRank <-cbind(EffRank, EffectiveRank)


  # ln lambda
  lnEigenVal<-log(EigenVal)

  # regression
  Line<-lm(lnEigenVal[1:50] ~ RankE)

  # plot line, a=intercept, b=slope
  abline(a=coef(Line)[1], b=coef(Line)[2], col="red")

  Slope=coef(Line)[2]
  PermSlope<-cbind(PermSlope, Slope)

  cat("\014")

  print(i)

}
## [1] 1…
## [1] 5000
abline(v = mean(EffRank), lty = 2, col="red",lwd = 2)
abline(v= (mean(EffRank)-sd(EffRank)), lty=1, col="red", lwd=1)
abline(v= (mean(EffRank)+sd(EffRank)), lty=1, col="red", lwd=1)
#calculate and print st deviation and mean for each parameter
RankCompression<-(mean(EffRank)/iEffectiveRank)

cat("Sum of Variance: ",iEigenSum)
## Sum of Variance:  0.001179661
cat("Effective Rank: ", iEffectiveRank)
## Effective Rank:  20.09512
cat("Permuted Effective Rank Mean: ", mean(EffRank))
## Permuted Effective Rank Mean:  45.66978
cat ("Permuted Effective Rank SD: ", sd(EffRank))
## Permuted Effective Rank SD:  0.3709488
cat("Rank Compression Ratio piRe/Re: ", RankCompression)
## Rank Compression Ratio piRe/Re:  2.27268
#Begin eigenvalue regression

# ln lambda

RankE<-seq(from=1, to=length(EigenTrim), by=1)

lnEigenTrim<-log(EigenTrim)

# regression
LineFit<-lm(lnEigenTrim ~ RankE)
DecaySlope<-coef(LineFit)[2]

cat("Decay Slope: ", DecaySlope)
## Decay Slope:  -0.1321483
cat("Slope Ratio, actual/permuted: ", DecaySlope/mean(PermSlope)  )
## Slope Ratio, actual/permuted:  2.704798
lniEigenVal<-log(iEigenVal)
## Warning in log(iEigenVal): NaNs produced
RankE<-seq(from=1,to=ERRoundUp,by=1)
iline<-lm(lniEigenVal[1:ERRoundUp]~RankE)
summary(iline)$r.squared
## [1] 0.927976
# plot line, a=intercept, b=slope
abline(a=coef(iline)[1], b=coef(iline)[2], lwd=2)
abline(v=iEffectiveRank, lty = 2, col="black",lwd=2)
```

```
#Begin Vrel calculation
EigenTrim<-iEigenVal[1:26]
meanEigen<-(sum(EigenTrim))/26
diffEigen<-(EigenTrim)-meanEigen
sqdiffEigen<-(diffEigen)^2
SumSqDifEig<-sum(sqdiffEigen)
VaRel<-(SumSqDifEig)/(((meanEigen)^2)*650)
cat("Vrel26: ", VaRel)
## Vrel26:  0.06878157
Vrel<-integration.Vrel(GPALMData,phy = NULL)
cat("VrelGeo: ",(Vrel$Re.obs))
## VrelGeo:  0.07792601
#plot function for bootstrapped rank and confidence interval;
#entered manually from bootstrap code

#abline(v = 15.564, lty = 2, col="black",lwd = 2)
#abline(v= (15.564 + 0.623), lty=1, col="black", lwd=1)
#abline(v= (15.564 - 0.623), lty=1, col="black", lwd=1)
```
